# Comparative Study between Measurement Data and Treatment Planning System (TPS) in Small Fields for High Energy Photon Beams

**DOI:** 10.1155/2014/901436

**Published:** 2014-05-06

**Authors:** Khaled El Shahat, Aziza El Saeid, Ehab Attalla, Adel Yassin

**Affiliations:** ^1^Radiation Oncology Department, Faculty of Medicine, Al Azhar University, Egypt; ^2^Biophysics Branch, Physics Department, Faculty of Sciences (Girls), Al Azhar University, Egypt; ^3^National Cancer Institute (NCI), Cairo University, Egypt

## Abstract

To achieve tumor control for radiotherapy, a dose distribution is planned which has a good chance of sterilizing all cancer cells without causing unacceptable normal tissue complications. The aim of the present study was to achieve an accurate calculation of dose for small field dimensions and perform this by evaluating the accuracy of planning system calculation. This will be compared with real measurement of dose for the same small field dimensions using different detectors. Practical work was performed in two steps: (i) determination of the physical factors required for dose estimation measured by three ionization chambers and calculated by treatment planning system (TPS) based on the latest technical report series (IAEATRS-398) and (ii) comparison of the calculated and measured data. Our data analysis for small field is irradiated by photon energy matched with the data obtained from the ionization chambers and the treatment planning system. Radiographic films were used as an additional detector for the obtained data and showed matching with TPS calculation. It can be concluded that studied small field dimensions were averaged 6% and 4% for 6 MV and 15 MV, respectively. Radiographic film measurements showed a variation in results within ±2% than TPS calculation.

## 1. Introduction

Human tissue is considered similar to water in terms of its physical properties as it represents 90% of its contents and how it interacts with radiation. For this reason, radiation doses (energy imparted per mass) prescribed to cancer patients typically refer to doses in water. Fundamental to small field dosimetry is the difficulty of linking the measurement in the clinic to that at the international standards laboratory so that the measurement signal is converted to absorbed dose in water [1]. The present aim in radiation therapy is to ensure that the uncertainty in the dose received by a patient does not exceed 5%. Considering all sources of uncertainty, this means that the dose at the calibration point of a linac has to be known to be within 2%.

The definition of a small field in radiation dosimetry is currently very subjective and ad hoc. There is no clear consensus definition as to what constitutes a small field. Commonly, a field size of less than 3 × 3 cm^2^ is considered outside the conventional treatment field size that needs special attention both in dose measurements and in dose calculations. A more scientific approach is needed to set the criteria which define a small field condition based on the beam energy and the density of the medium [2].

There are essentially three “equilibrium factors” that determine the scale of the field as small field it is consideration as small field or not:

- the dimensions of beam the same as projected upon the detectors,
- suitable dimension of detector used in measurements,
- small scattered radiation due to small field dimensions.

Small field dosimetry plays an important role in modem radiotherapy for many reasons. Treatment planning system commissioning requires the input of beam data, specific to the treatment units. This requires the acquisition of depth dose profiles, beam profiles, and RDFs, as introduced in the previous sections [3].

The difficulty in achieving accurate small field dose measurements is similar to the factors which affect specific measurements in the dosimetry of larger fields (e.g., the steep dose gradients in penumbral regions and the loss of charged particle equilibrium in the build-up region) but they are accentuated. The loss of lateral charged particle equilibrium generally results in a decreased dose at central axis, rather than just at the beam edges and in the build-up regions. In addition, the narrowing of the beam results in a more peaked lateral dose profile, which escalates the requirement for higher spatial resolution not only in the penumbral regions but also at the central axis.

## 2. Instrumentation

The Linear accelerator used was Elekta model—dual energies 6 and 15 MV photon beam and four electron energies (6, 8, 10, and 15 MeV).The treatment planning system used in this work is Precise Plan. The ionization chambers used were Farmer ionization chamber, vented cylindrical ionization chamber for measuring high-energy photon and electron radiation in water or in solid-state material, Semiflex chamber (0.125 cm³), and Pinpoint chamber (0.015 cm³). The type of waterproof chambers that has been specially designed for relative beam profile measurements was done by motorized water. The Pinpoint chamber is ideally suited for measurements of small fields of inner diameter 2.9 mm. Farmer dosimeter model (2570/1B (# 1164)) andradiographic Filmmodel Kodak X-OmatV.

## 3. Methods

The absorbed dose was determined after the mechanical check to ensure the suitability of the machine to perform the dosimetric measurements to ensure the suitability of the machine to perform the dosimetric measurements. The laser lines that compromise the cross wires in the light field area should be checked. The isocentre point for gantry, collimator, and couch rotation should be checked to ensure. Then, adjust the water phantom at 100 cm SSD and locate the ionization chamber at 10 cm depth for 6 and 15 MV photon beam with 90-degree gantry angle, zero-degree collimator angle, and zero-degree couch angle according to IAEA protocol (TRS 398).

Measure the pressure and temperature to calculate the factor *K*_*T*,*p*_ (correction factor temperature and pressure) which estimate the effect of pressure and temperature on measurement.

When different small fields by field were irradiated to measure absorbed dose for each field. The data carried out by TPS were compared with practical data of ICs.

*Estimating the standard film to be the reference dose gradient by irradiating different films to gradual from 20 to 100 monitor units. Where the irradiated film placed in the percpix sheets placed at the depth of maximum dose for each energy (1.6 cm for 6 MV and 2.7 cm for 15 MV), process the film and draw an isodose curve through which we can determine the absorbed dose for each irradiated film optical density value can be determined.*

When irradiating different fields, determining the absorbed dose for each field, and comparing the results with the TPS data.

## 4. Results and Discussion

### 4.1. Absolute Values of Dose Measurement by Different Ionization Chambers

#### 4.1.1. Small Field Area for 6 MV

The calculated data of absorbed dose taken by the precise treatment planning system versus the measured data for the three ionization chambers were represented in Table 1; the three types of ionization chambers (Farmer, Pinpoint, and Semiflex) were used for different dimensions at reference depth of 10 cm with respect to small field dimensions for 6 MV.

**Figure 1:**
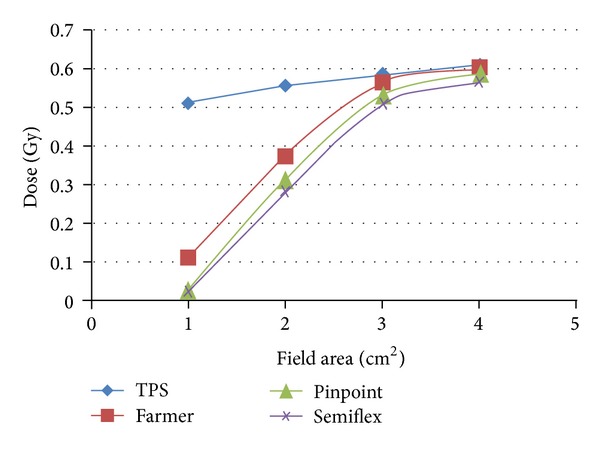
TPS data versus the measured values by different ICs.

Figure 1 shows the comparison results of calculated data by TPS and measured values for 6 MV for different small field dimensions starting from 1 × 1 to 4 × 4 cm^2^ dimensions at 100 cm SSD, at reference depth of 10 cm in a standard phantom.

The comparison of the TPS calculated data and measured parameters by different ionization chambers described the difference between calculated and measured data which were observed in three regions. The first one represents the difference between the calculated data by TPS and measured data by ionization chambers data for two small field dimensions (3 × 3 and 4 × 4 cm^2^) were averaged to 6% for all Ionization chambers used in the current study. The second region shows the difference averaged to 41% between the calculated data by TPS and measured data by ICs for 2 × 2 cm^2^ field dimensions. The third region illustrates discrepancy exceeding the average of 80% between the calculated data by TPS and measured data by ICs for 1 × 1 cm^2^ field dimensions.

The measured doses in standard phantom were less than the TPS values for small field dimensions, less than 3 × 3 cm^2^; this underestimation is due to electronic equilibrium which is not sufficient for fully scattered photons from linear accelerator of photon beam energies 6 MV [4].

#### 4.1.2. Small Field Area for 15 MV

The calculated data of absorbed dose taken by the precise treatment planning system versus the measured data for the three ionization chambers were presented in Table 2; the three types of ionization chambers (Farmer, Pinpoint, and Semiflex) were used for different field dimensions at reference depth of 10 cm relative to small field dimensions for 15 MV.

**Figure 2:**
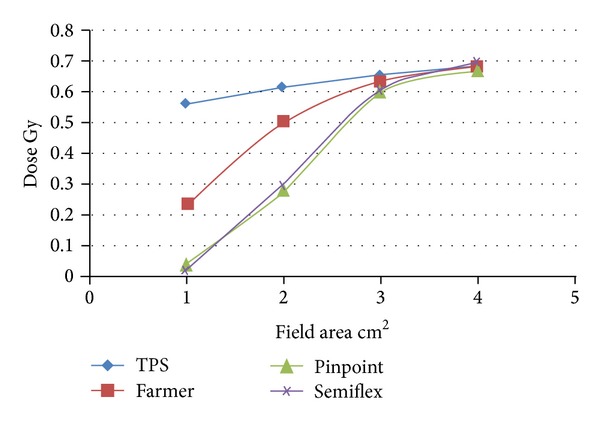
TPS data versus the measured values by different ICs.

Figure 2 shows the results of comparison for calculated data by TPS and measured results concerning 15 MV for different small field dimensions starting from 1 to 4 cm^2^ dimensions at 100 cm SSD, at reference depth of 10 cm in a standard phantom.

The discrepancies between calculated data and measured values which were observed in two regions are illustrated as follows. The first one represents difference between the calculated data by TPS and measured data by ICs for small field dimensions, (3 × 3 and 4 × 4 cm^2^) averaged to 4% for the three types of ICs used in present work. These data are in agreement with data published by [5] with respect to average variation 3.8%. The second region illustrates discrepancy which exceeds the average by 49% between TPS and measured data by using ICs for field dimensions (1 × 1 and 2 × 2 cm^2^). The measured doses in standard phantom were less than the TPS values for small field dimensions, less than 3 × 3 cm^2^; this underestimation is due to electronic equilibrium which is not sufficient for fully scattered photons from linear accelerator of photon beam energies 6 and 15 MV [4].

### 4.2. Absolute Values of Dose Measurement by Radiographic Film (RF)

#### 4.2.1. Small Field Area for 6 MV

The calculated data of absorbed dose taken by the precise treatment planning system versus the measured data by radiographic film were represented in Table 3 for different small field dimensions (1, 2, 3, and 4 cm^2^) at *D*_max⁡_ (depth for measured max dose) 1.6 cm at 50 Mu.

**Figure 3:**
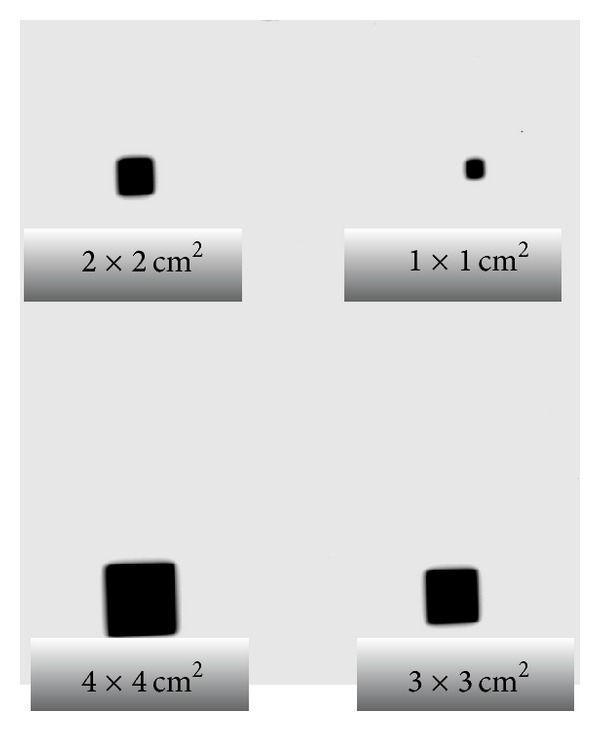
Radiographic film exposed to 6 MV for various fields.

Figure 3 shows the optical density for different small fields measured at *D*_max⁡_ 1.6 cm, 100 cm SSD, and 50 Mu in solid phantom.

**Figure 4:**
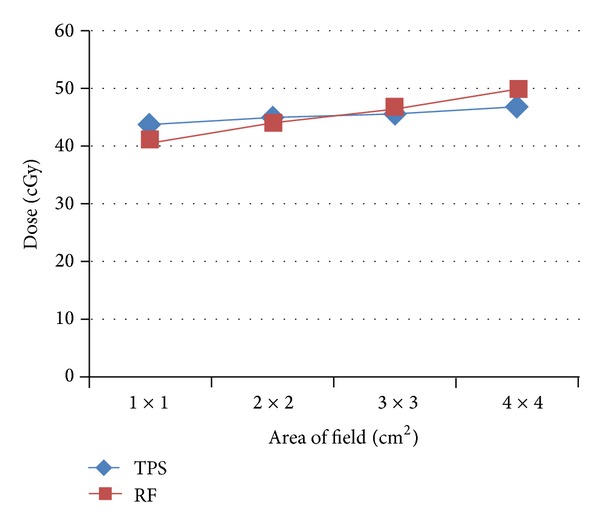
TPS values against the measured data by radiographic film.

Figure 4 indicates mild differences between the TPS and the experimental data for fields 2 × 2 and 3 × 3 cm^2^; this difference within ±2% is due to good accuracy of measurement planning system. These data are in agreement with results obtained by [6], the change being within ±1.5%.

For field area 1 × 1 cm^2^, the measured data was decreased within 7% and, for field 4 × 4 cm^2^, the measured data increased within 6%. This is due to the quantity of scattered radiation which is directly proportional to field area.

#### 4.2.2. Small Field Size for 15 MV

The calculated data of absorbed dose taken by the precise treatment planning system versus the measured data by radiographer film were tabulated in Table 4 for different small field dimensions (1, 2, 3, and 4 cm^2^) at *D*_max⁡_ 2.7 cm and 50 Mu.

**Figure 5:**
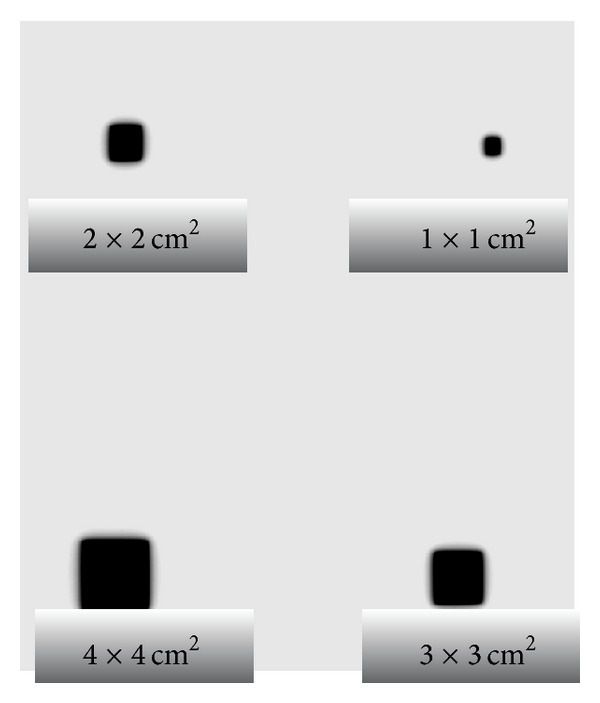
Radiographic film exposed to 15 MV for various fields.

Figure 5 illustrates the optical density for small fields at *D*_max⁡_ 2.7 cm, 100 cm SSD, and 50 Mu in solid phantom.

**Figure 6:**
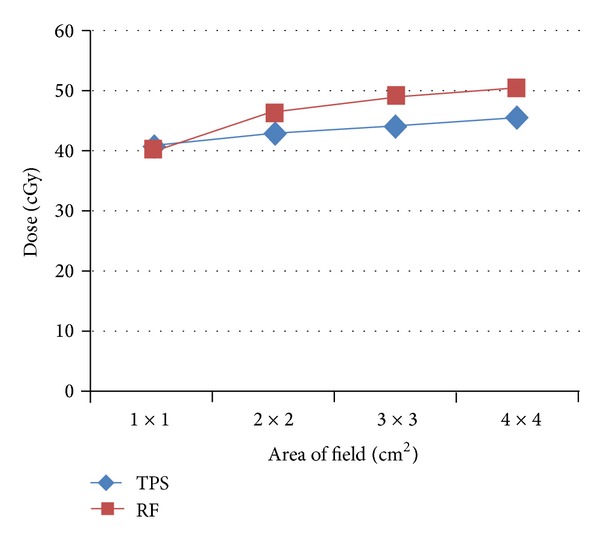
TPS values against the measured data by radiographic film.

Figure 6 shows small difference between the TPS and the experimental data using radiographic film; the results were the same for TPS and measured data, at only field dimension 1 × 1 cm^2^. Measured data was increased as field area enlarged from 2 × 2 to 4 × 4 cm^2^ by constant value of 5% [7, 8]. Film response error includes processing procedure parameters and energy dependent upon non-linear response. Corrections were applied for response deviations due to the energy dependence, including response changes due to field area and depth. Processing variation errors were minimized by using a reference calibration film exposed and processed with each experiment. The response relationship between the single film calibration and multifilm central axis were determined [9].

## 5. Conclusion

- The measured doses in standard phantom were less than the TPS values for small field dimensions; for fields less than 3 × 3 cm^2^, this underestimation was due to electronic equilibrium which is not sufficient for fully scattered photons from linear accelerator of photon beam energies 6 and 15 MV. On the other hand, field dimensions more than 3 × 3 cm^2^ have an average of 6% for three types of ionization chambers (Farmer, Pinpoint, and Semiflex) used in the current study to 6 MV while 15 MV shows an average of 4% for three types of ionization chambers.
- The measured data by radiographic film for 6 MV shows mild differences between the TPS and the experimental data for fields 2 × 2 and 3 × 3 cm^2^; this difference within ±2% is due to good accuracy of measurement planning system. For field area 1 × 1 cm^2^, the measured data was decreased within 7% and for field 4 × 4 cm^2^ the measured data increased within 6%. This is due to the quantity of scattered radiation which is directly proportional to field area.
- Small difference existed between the TPS and the experimental data using radiographic film for 15 MV; the results were the same for TPS and measured data, at only field dimension of 1 × 1 cm^2^. Measured data was increased as field area enlarged from 2 × 2 to 4 × 4 cm^2^ by constant value of 5%.

## Figures and Tables

**Table 1:** TPS data versus the measured values by different Ionization chambers for 6 MV.

Field area cm^2^	TPS Gy	Farmer Gy	Pinpoint Gy	Semiflex Gy
1 × 1	0.514	0.111	0.025	0.022
2 × 2	0.557	0.375	0.315	0.281
3 × 3	0.585	0.565	0.532	0.511
4 × 4	0.608	0.603	0.588	0.565

**Table 2:** TPS data versus the measured results by different Ionization chambers for 15 MV.

Field area cm^2^	TPS Gy	Farmer Gy	Pinpoint Gy	Semiflex Gy
1 × 1	0.564	0.235	0.042	0.023
2 × 2	0.617	0.503	0.282	0.299
3 × 3	0.655	0.637	0.598	0.607
4 × 4	0.686	0.682	0.669	0.698

**Table 3:** TPS versus the measured results by radiographic film for 6 MV.

Field area cm^2^	TPS cGy	RF cGy
1 × 1	43.9	40.67
2 × 2	44.65	43.8
3 × 3	45.65	46.35
4 × 4	46.56	49.83

**Table 4:** TPS versus the measured data by radiographic film for 15 MV.

Field area cm^2^	TPS cGy	RF cGy
1 × 1	40.9	40.25
2 × 2	42.71	46.55
3 × 3	44.15	49.19
4 × 4	45.65	50.65
